# Selecting representative model micro-organisms

**DOI:** 10.1186/1471-2180-5-26

**Published:** 2005-05-17

**Authors:** BR Holland, J Schmid

**Affiliations:** 1 Allan Wilson Centre for Molecular Ecology and Evolution, Massey University, Palmerston North, New Zealand; 2 Institute of Molecular BioSciences, Massey University, Palmerston North, New Zealand

## Abstract

**Background:**

Micro-biological research relies on the use of model organisms that act as representatives of their species or subspecies, these are frequently well-characterized laboratory strains. However, it has often become apparent that the model strain initially chosen does not represent important features of the species. For micro-organisms, the diversity of their genomes is such that even the best possible choice of initial strain for sequencing may not assure that the genome obtained adequately represents the species. To acquire information about a species' genome as efficiently as possible, we require a method to choose strains for analysis on the basis of how well they represent the species.

**Results:**

We develop the *Best Total Coverage *(BTC) method for selecting one or more representative model organisms from a group of interest, given that rough genetic distances between the members of the group are known. Software implementing a "greedy" version of the method can be used with large data sets, its effectiveness is tested using both constructed and biological data sets.

**Conclusion:**

In both the simulated and biological examples the greedy-BTC method outperformed random selection of model organisms, and for two biological examples it outperformed selection of model strains based on phylogenetic structure. Although the method was designed with microbial species in mind, and is tested here on three microbial data sets, it will also be applicable to other types of organism.

## Background

To gain insight into biological processes, biologists often rely on model organisms. Focusing the funding and research efforts of many research groups on one model organism is considered more likely to advance the field than scattering the same resources over a large number of organisms. A recent application of this philosophy is in genome sequencing: rather than simultaneously initiating the sequencing of the genomes of many individuals from a species, typically a single representative is chosen. In microbiology the initial choice of model strain is frequently a well-characterized laboratory strain, often selected at a time when the ability to determine population structure and to measure genetic distances was limited (see for example [1,2]). There is merit in choosing a physiologically well-characterised laboratory strain for genome sequencing, as it facilitates the interpretation and annotation of sequence data. However, it has often become apparent that the model strain initially chosen does not represent important features of the species [3-5]. For micro-organisms, the diversity of their genomes is such that even the best possible choice of initial strain for sequencing may not assure that the genome obtained adequately represents the species [5-7]; additional strains may need to be studied [7]. To acquire information about a species' genome as efficiently as possible, we require a method to choose strains for analysis on the basis of how well they represent the species.

The problem of choosing representative model strains is complicated by the fact that the value of an organism as a representative depends on the features it is meant to represent. As an example illustrating this problem, assume that we require model organisms that represent a larger group of organisms in terms of amino acid sequence at a particular open reading frame (ORF). Suppose that we are able to find a single model organism which represents the entire group; i.e. BLAST searches find significant homologies between the amino acid sequence of the model protein and proteins from the other organisms. Let us assume that homologies between the protein of the model organism and the more distant members of the group are low, albeit still significant. We would conclude, using the BLAST homology criterion that we have represented the group, and have done so efficiently, using only one model organism. However, if we were then to try to use this same organism to design PCR primers for amplifying the ORF in all members of the group, the model organism would fail for the more distant members. Likewise if we were to investigate another, less conserved ORF across the group, the model organism may fail to show significant homology to a large number of members of the group even at the amino acid sequence level. Another difficulty in choosing model organisms is that our selection will be based on limited and biased information obtained in a screen of the group we wish to represent, such as sequences from a few genes or restriction fragment length polymorphism (RFLP) data which may not represent, or only be loosely linked to, diversity across the entire genome.

In this paper we describe and evaluate approaches for the rational selection of model organisms, which take these problems into consideration.

## Results and discussion

### Finding the best model organism

As illustrated in the introduction, there will be some threshold distance, i.e. level of genetic difference, between the model organism and other organisms, above which the model organism will no longer be a useful representative, and different applications will have different threshold values. If we need a model organism for some specific application where a threshold distance, *T*, is known, the problem of selecting the best model organism can be solved by choosing the organism for which the greatest number of other organisms lie within this genetic distance, *T*. We refer to this criterion as *Best Coverage*.

**Figure 1:**
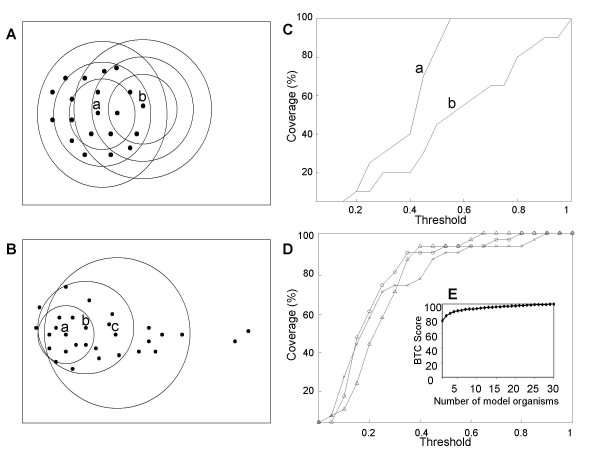
**Coverage plots for example data sets. **A and B) Artificial data sets where organisms are represented by dots with Euclidean distances. The concentric circles around the labelled organisms indicate how many organisms would be well represented by this choice of model strain for different threshold values. C and D) Coverage plots for the data sets in A and B respectively, showing how many organisms are well represented for increasing threshold values. Key for D: **a **– x, **b **– o, **c **– Δ. E) *Best Total Coverage *score (as a percentage of maximum) for 1–30 model organisms, for data set B, the model organisms are selected using a greedy implementation of the BTC method as described later in the text.

For the purpose of illustration we display a constructed example where distances between organisms are the distances on a plane (Figs. 1A and 1B). Intuition suggests that model organisms chosen according to the *Best Coverage *criterion will be central to the group of interest. For example, in Figure 1A organism **a **is the most central, it has the best coverage for any choice of threshold distance. Conversely, a non-central organism such as organism **b **has worse coverage for any value of *T*. Choosing a model organism in the example in Figure 1A is trivial because the members of the group are distributed in a highly symmetrical fashion. However, in many cases members of a group will not be symmetrically distributed and there will be no obvious central organism. In such a case, shown in Figure 1B, the value of the threshold distance, *T*, will affect the decision as to which organism is best. Depending on the choice of *T*, organism **a**, **b**, or **c **could be preferred. Figures 1C and 1D display *Coverage *for increasing values of *T *for the marked organisms in 1A and 1B respectively. Note that in Figure 1D organism **a **is best for low threshold values and that organism **c **is best for high threshold values, however, taken as an aggregate over a range of T values organism **b **is superior.

Finding the best threshold distance for selecting model organisms from a group poses a number of difficulties. Firstly, as noted, different applications require different thresholds and so no one threshold distance will be ideal as a basis of model strain selection, unless the model strain is only to be used for one particular application. Secondly, even if we knew in advance the exact feature which we want the model organism to represent, there will only be limited information on the variation in this feature for the entire group of organisms -after all, if the organisms were exactly characterised there would be no need to pick model organisms for further study. Lastly, if we want to make a good choice of model organisms we will need to initially sample a large number of members of the group the model organisms are supposed to represent. This favours the use of methods that produce "quick and dirty" estimates of relationships between members such as short sequence alignments, fragment length polymorphism, biotyping or enzymatic activity. The resulting distances will only be a rough guide to the true relationship between organisms, and their relationship to *T *for specific applications will be unknown.

These considerations suggest that a good criterion for choosing a model organism is that it be representative for a range of *T *values. This can be implemented by normalising the available distances within a group to be represented to have a maximum of 1, and summing the *Coverage *criterion for *T *at steps between 0 and 1, e.g. {0 0.05, 0.10... 0.95, 1.00}. We refer to this score as being the *Total Coverage *for the model organism, as it is an aggregate over a range of *T *values. The *Best Total Coverage *(BTC) method picks the organism for which this score is largest. Relating this to the coverage graphs (Figs. 1C and 1D) the BTC method picks the organism with the largest area beneath the curve, for 1C this is organism **a**, and for 1D it is organism **b**.

### Determining the number of model organisms required

We can see in Figure 1A, and especially 1B, that for small threshold values, even the best possible model organism, as selected by the best total coverage method, cannot represent the entire group of organisms. Rather than choosing a single model organism we would prefer, if possible, to choose a set of model organisms so that as many organisms as possible lie within the threshold distance of at least one model organism for a particular application. Obviously coverage will continue to improve, or at least not get worse, as more and more model organisms are allowed. Indeed, in the ideal case every unique organism would be chosen as a model organism. However, financial and time constraints usually mean that such a solution is not feasible. Plotting the increase in coverage for different numbers of models organisms allows one to judge when no significant improvement results from adding another model organism, or whether the improvement outweighs the cost of dealing with an extra model organism. For example, Figure 1E shows the BTC score (as a percentage of the maximum possible) for the data set in Figure 1B with from one up to thirty model organisms allowed. In this example the graph suggests that only marginal improvements in coverage can be obtained by having more than one or two model organisms. In the case where there are distinguishable clusters of genetically similar strains, the value of k could be predetermined based on the number of clusters.

### Computational strategies for implementing the methods

For practical application of the strategies outlined above, we must consider the computational complexity of finding the best set of model organisms. For a group of *n *isolates and a predetermined number of model organisms *k *there are *n*!/*k*!(*n *- *k*)! possible sets of model organisms to be tested, and for each set of model organisms it requires *nk *operations to calculate *Coverage*, because for each of the *n *isolates, at worst *k *putative model organisms must be checked to determine if the distance between the isolate and the model organism is less than *T*. So, for a fixed *k*, it will take time proportional to *n*^*k *+ 1 ^to compute the best set of model organisms. This means that for large *n *and *k *it will become computationally intractable to test all possible sets of model organisms. For example, for *k *= 3 model organisms from a set of 100 organisms there are 161,700 possible sets of model organisms to consider, for *k *= 5 model organisms there are 75,287,520 sets to consider, and for *k *= 10 there are 1.73 × 10^13^.

To avoid this problem a greedy approximation to the exact method can be used. Initially one model organism is chosen, the one that gives the largest *Total Coverage *score, then model organisms are added one at a time, at each stage picking the organism which gives the largest improvement in the *Total Coverage *score, until some predefined number of model organisms have been selected. We name this the greedy-BTC method.

### Test of the effectiveness of the greedy-BTC method for choosing model organisms

We tested the performance of the greedy method in several ways. Firstly we compared the greedy-BTC method against the exact-BTC method using a range of simulated sequence data (see the Methods section for details on data generation). The greedy method gave similar results to the exact method for problems with small (computationally feasible) numbers of model sequences (Table 1; for comparison the *Total Coverage *scores for randomly selected model sequences are also included). For larger numbers of model organisms we would expect that the greedy method would perform less well relative to the exact method. However, in this context we are not so concerned with finding an optimal solution – a good solution will do.

**Figure 2:**
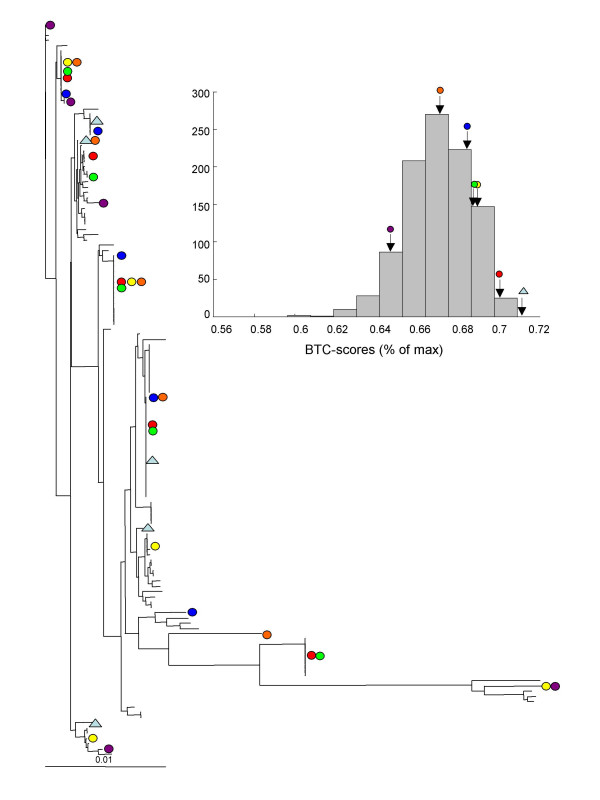
**Comparing greedy-BTC model strains to researchers' selections for the *Enterococcus *data. **Neighbor-joining [10] tree based on MLST data for 139 *E. faecium *strains. Each MLST scheme has seven genes, these genes were concatenated together and a distance matrix (used by both neighbor-joining and the greedy-BTC method) was constructed based on uncorrected distances between pairs of sequences (i.e. the proportion of sites that differ). The blue triangles indicate the five model strains selected using the greedy-BTC method. Coloured circles indicate model strains selected by six researchers. The inset histogram compares the BTC-scores of the greedy and human selection to 1000 random selections of five model strains.

**Figure 3:**
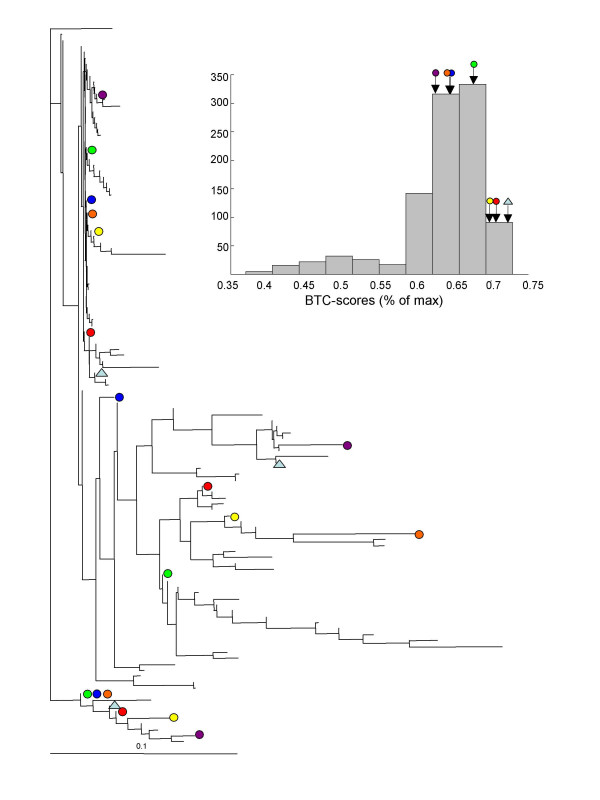
**Comparing greedy-BTC model strains to researchers' selections for the *Candida *data. **Neighbor-joining tree based on MLST data for 121 *C. albicans *strains (see figure 2 caption for details of construction). The blue triangles indicate the three model strains selected using the greedy-BTC method. Coloured circles indicate model strains selected by six researchers. The inset histogram compares the BTC-scores of the greedy and human selection to 1000 random selections of three model strains.

Secondly we compared the greedy-BTC method against both a large number of random selections of model organisms and researchers' selections based on phylogenetic evidence. We used two MLST data sets: 139 strains of *Enterococcus faecium *[8], and 121 strains of *Candida albicans *[9]. Six researchers (four phylogenetics researchers from the Allan Wilson Centre for Molecular Ecology and Evolution, and the two authors) were given copies of the two trees and asked to select three and five model strains respectively that they felt would be good representatives of the group of strains based on the phylogenetic tree. (The reasons we fixed *k = 5 *for the *E. faecium *data and *k = 3 *for the *C. albicans *data are detailed in a later section.) The trees in figures 2A and 3A show the researchers' selections of model organisms and the greedy-BTC choice of model organisms. Figures 2B and 3B compare the performance of these selections along with 1000 random selections of model organisms. The greedy-BTC strains performed better than random selection in all but 4/2000 cases and always outperformed the human selections.

An interesting outcome of this experiment is that strain selections which seem almost identical based on the phylogenetic tree, for example those selections represented by red and green circles in figure 3A, performed quite differently. This is probably because trees cannot (except in the case of perfectly treelike distances) reflect all of the information in the distance matrix [10].

### Assessing usefulness of the method in choosing model organisms representing characters unknown at the time of selection

As pointed out in the introduction, selection of model organisms will often be based on characters different from the ones which will later be studied using the model organism. Since different loci may evolve according to different processes in different lineages [10], selecting a model organism using one set of characters does not guarantee that it is representative for other characters. The exchange of genetic material between organisms of different species (horizontal gene transfer) will produce additional problems of a similar nature. The true test of a method for selecting model organisms is therefore if it can arrive at a model organism which is reasonable representative for characters not used in its selection.

We therefore tested the BTC method on three examples of micro-biological data sets in which we could assess the representativeness of model organisms for characters not used in their selection. In each case we began by using one character set to choose the greedy-BTC model strains for *k *in the range 1–10. As in the previous example with simulated data we compared the greedy-BTC score to the *Total Coverage *score attained by random sets of model organisms for each data set and value of *k*. Then, for a fixed value of *k*, we compared our choice of model organisms with random choices of *k *organisms, in terms of how well they represented the characters not used in the selection of the model organisms.

**Figure 4:**
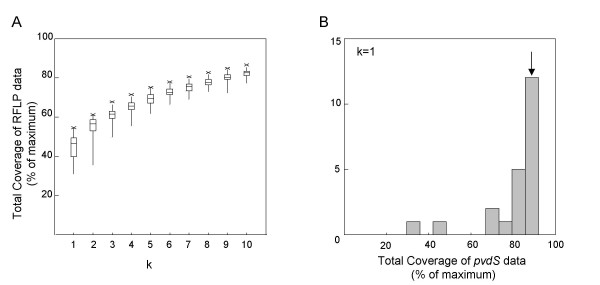
**Performance of the greedy-BTC method for *Pseudomonas *data. **A) The greedy *Best Total Coverage *score for *k *= 1–10 model organisms, for the *Pseudomonas *RFLP distances, is shown by the crosses. For each value of *k *the box and whisker plot indicates the quartiles (i.e. the box indicates those 50 % of the values closest to the median) and range for 1000 random choices of model organisms. *Total Coverage *scores were produced by adding up the percentage of organisms represented at each of 20 threshold value intervals (0, 0.05, 0.10,...,0.95 and 1.0); they are reported as a percentage of the maximum possible *Total Coverage *score. B) Histogram showing the *Total Coverage *scores of 1000 random choices of model organism, the arrow indicates the *Total Coverage *score for the BTC choice of model strain.

The first example was a sample of 22 *Pseudomonas aeruginosa *strains, for which both RFLP typing data and sequence data (*pvdS *gene) are available [11]. We used the RFLP data for model strain selection. As expected, the plot of greedy BTC score versus *k *for the RFLP based distances (Fig. 4A) showed a gradual improvement in *Total Coverage *as increasing numbers of model strains are used. For each value of *k *(*k *= 1–10) we compared the *Total Coverage *of the greedy-BTC model strains to the *Total Coverage *of 1000 randomly chosen sets of *k *strains. For all values of *k *tested the greedy-BTC score is in the top quartile of the distribution of scores for randomly chosen strains.

To assess the representativeness of the BTC model strain on the *pvdS *data (not used in model strain selection) we fixed *k *= 1, as the total number of strains was fairly small, and Figure 4A did not suggest any obvious clustering in the data (clustering would be indicated by a sharp jump in coverage for some value of *k*). We then measured the *Total Coverage *of the selected model strain for the *pvdS *gene and compared it to the *Total Coverage *of the remaining 21 strains (Fig 4B). (In order to compute the *Total Coverage *score for the *pvdS *data we constructed Hamming distances from the sequence alignment.) In 55% of cases random selection did equally well (this was unsurprising as 12/22 of the strains had identical *pvdS *sequences), but in 45% of the cases random choice gave a worse representation than the BTC selected model strain.

The second data set consisted of partial sequences of seven genes (*adk, atpA, ddl, gyd, gdh, purK*, and *pstS*) of 139 *Enterococcus faecium *strains [8]. We constructed seven test data sets, for each a distance matrix was generated by taking the Hamming distances from the sequences for six of the loci (i.e. each time we omitted one locus). Using these distances we carried out selection of model organisms separately for each of the seven test data sets. We then tested the performance of the greedy-BTC method by assessing how well the model organisms represented the seventh locus (omitted when constructing the distance matrix).

**Figure 5:**
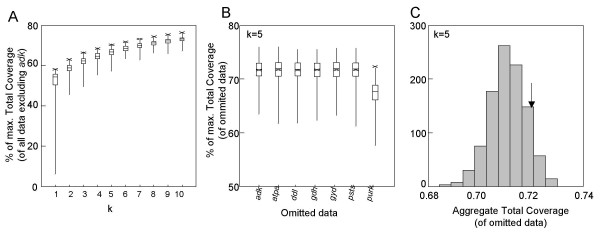
**Performance of the greedy-BTC method for *Enterococcus *data. **A) The greedy *Best Total Coverage *score for *k *= 1–10 model organisms, for the distances generated from all loci excluding *adk*, is shown by the crosses. For each value of *k *the box and whisker plot indicates the quartiles (i.e. the box indicates those 50 % of the values closest to the median) and range for 1000 random choices of model organisms. *Total Coverage *scores were produced by adding up the percentage of organisms represented at each of 20 threshold value intervals (0, 0.05, 0.10,..., 0.95 and 1.0); they are reported as a percentage of the maximum possible *Total Coverage *score. B) For each omitted locus the box and whisker plot indicates the quartiles and range for 1000 random choices of 5 strains. The score of the five greedy-BTC model strains are shown by crosses. C) Histogram showing the aggregate *Total Coverage *scores of 1000 random choices of five strains summed over the seven data sets, the arrow indicates the aggregate *Total Coverage *score for the BTC choices of model strains.

For each value of *k *(*k *= 1..10) we compared the *Total Coverage *of the greedy-BTC model strains to the *Total Coverage *of 1000 randomly chosen sets of *k *strains. The results for one of these data sets (*adk *omitted) are shown in Figure 5A. The greedy-BTC model strains were always in the top quartile of the distribution of scores for random strains.

To assess the representativeness of the greedy-BTC model strains on the omitted gene (not used in model strain selection) we fixed *k *= 5, and compared the five greedy-BTC model strains to random selections of five strains. Figure 5B shows the performance on each data set. In one case the greedy-BTC model strains outperformed the random strains and in six cases the greedy-BTC model strains performed about equally to the median random score. However, note that the distribution of random scores is skewed – i.e. the best random sets of strains had scores about 5% higher than the greedy-BTC model strains but the worst random sets had scores about 10% lower than the model strains. Figure 5C shows the aggregate performance over all seven data sets. Picking strains systematically using the BTC method did better than picking a random set of five strains in 844 out of 1000 cases.

The third data set consisted of partial sequences of seven genes (*AAT1a*, *ACC1*, *ADP1*, *MPIb*, *SYA1*, *VPS13*, and *ZWF1b*) of 122 *Candida albicans *strains [9]. Similarly to the previous example, we generated seven distance matrices, each based on six of the loci, and, using these distances, carried out selection of model strains. We then tested our choice by assessing how well the model strains represented the alleles at the seventh locus (omitted when constructing the distance matrix).

**Figure 6:**
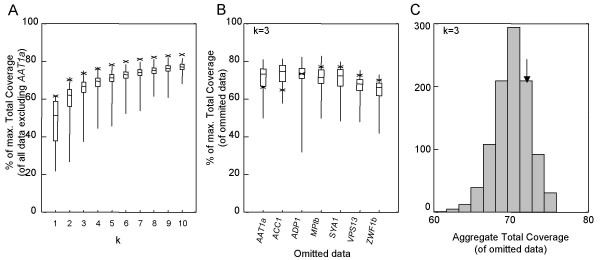
**Performance of the greedy BTC method for *Candida *data. **A) The greedy *Best Total Coverage *score for *k *= 1–10 model organisms, for the distances generated from all loci excluding *AAT1a*, is shown by the crosses. For each value of *k *the box and whisker plot indicates the quartiles (i.e. the box indicates those 50 % of the values closest to the median) and range for 1000 random choices of three strains. *Total Coverage *scores were produced by adding up the percentage of organisms represented at each of 20 threshold value intervals (0, 0.05, 0.10,..., 0.95 and 1.0); they are reported as a percentage of the maximum possible *Total Coverage *score. B) For each omitted loci the box and whisker plot indicates the quartiles and range for 1000 random choices of three strains. The score of the three greedy-BTC model strains are shown by crosses. C) Histogram showing the aggregate *Total Coverage *scores of 1000 random choices of three strains summed over the seven data sets, the arrow indicates the aggregate *Total Coverage *score for the BTC choices of model strains.

For each value of *k *(*k *= 1..10) we compared the *Total Coverage *of the greedy-BTC model strains to the *Total Coverage *of 1000 randomly chosen sets of *k *strains. The results for one of these sequence sets (*AAT1a *omitted) are shown in Figure 6A. The greedy-BTC model strains were always in the top quartile of the distribution of scores for random strains, and for *k *= 5, 6, 7, 8, 9, and 10 the scores for the greedy-BTC model strains were outside the range of scores for random sets of strains.

To assess the representativeness of the greedy-BTC model strain on the omitted gene (not used in model strain selection) we fixed *k *= 3, and compared the three greedy-BTC model strain to random selections of three strains. Figure 6B shows the performance on each data set. In four out of seven cases the greedy-BTC model strains outperform randomly chosen strains at representing the omitted gene, but for *AAT1a *and *ACC1 *random selection does better. Figure 6C shows the aggregate performance over all seven data sets, in 759 cases out of 1000 the greedy-BTC model strains outperformed the randomly chosen strains.

## Conclusion

The greedy-BTC method outperformed selections based on phylogenetic evidence made by researchers with experience in phylogenetics. By testing the greedy-BTC choice against the exact-BTC where feasible or against a large number of random selections of model strains we also demonstrated that the method comes close to achieving optimal representation of organisms (according to the BTC criterion).

The greedy-BTC also performed well in the more challenging situation where the characters to be represented were not used in model strain selection; for most of the biological data sets, with the exception of two loci in the *Candida albicans *data, the greedy-BTC model organisms did an equal or better job than random organisms of representing data not used in model strain selection.

One interesting feature of the distributions of *Total Coverage *scores for random organisms is its skewness – many sets produce good scores but there is a long tail of poor scores. This feature seemed to be consistent across a range of data sets. This means that picking organisms at random may occasionally do a little better than using the greedy-BTC method but it may also do a lot worse.

The three biological data sets analysed suggest that the BTC method we have developed should facilitate selection of model organisms that will be representative of the group of interest for a wide range of applications. Nevertheless, differences in the rate of evolution at different loci plus other phenomena such as horizontal gene transfer place limits on the degree of reliability of selected model organisms in terms of representativeness in regard to other, unknown loci. We would therefore suggest that if the BTC method were used to select organisms for a project associated with major expenses (in terms of time or money), such as a genome sequencing project, it would be advisable to begin by eliminating part of the input data and testing how well the method works at selecting model organisms that capture the diversity of the omitted part of the data. Such an analysis will not only provide a general idea of how well the selected organisms will represent the sample, it will also reveal if some of the markers intended for selection may give misleading information. If, for instance one of them had been acquired by horizontal gene transfer, this should become apparent as poor representation of allelic diversity at that marker locus by model organisms selected on the basis of the remaining markers.

We note that one problem our method does not address is how to obtain a suitable collection of the group of organisms on which the BTC method is to be used to choose representatives. The BTC method is aimed at optimally representing a group defined by its user, and highly prevalent genetically similar subgroups in this user-defined group will carry more weight than low prevalence groups. Therefore, if the group is biased so that particular groups are over represented, the BTC choice of model strains will be biased as well.

## Methods

The greedy-BTC and exact-BTC methods described for selecting model organisms have been implemented in a C program that has been tested using both UNIX and WindowsXP. The exact method is only feasible for k ≤ 3 unless the total number of strains is also fairly small; the program automatically defaults to the greedy method if k ≥ 4 and n ≥ 10. The program also works for user-defined threshold values. All code is available on request from b.r.holland@massey.ac.nz.

The method described can be expressed mathematically as follows. Firstly let *X *be the set of all organisms, and *d(m,x) *a measure of the distance between organism *m *and *x*. Define *y(m,x) *= 1, if *d(m,x) *<*T*,

*y(m,x) *= 0, otherwise.

In the case of choosing a single model organism *m*



To solve the multiple model organism case we choose a set *M *of fixed size *k*, such that



The test data used to generate Table 1 was generated using the software Treevolve version 1.3.2 [12] to simulate DNA sequences along a large (1000 taxon) random tree according to a simple model of nucleotide substitution (the Jukes Cantor model [13]). One hundred data sets of size 20 were then sampled from this large data set, and distance matrices were generated by taking the uncorrected distance between sequences (this is the proportion of sites that differ between the two sequences).

## List of abbreviations

BTC – best total coverage

MLST – multi-locus sequence typing

ORF – open reading frame

PCR – polymerase chain reaction

RFLP – restriction fragment length polymorphism

## Authors' contributions

B.R.H. developed and implemented the methods, performed simulations and wrote the bulk of the manuscript. J.S. developed the methods, wrote the introduction section of the manuscript, edited the manuscript, and sourced the example data sets.

## Figures and Tables

**Table 1:** Performance of the greedy algorithm in selecting model organisms from simulated DNA sequence data sets.

	Total coverage (percent of exact method)		
	*k *= 1	*k *= 2	*k *= 3

Greedy Score	100.0%	99.6%	99.6%
Random Score	77.7%	76.5%	76.4%

The scores are the mean *Total Coverage *over the 100 simulated data sets for *k *= 1, 2 or 3 with the greedy and random scores (scores obtained when randomly chosen sequences are used as model strains) expressed as a percentage of the exact scores. All data sets were generated as described in the methods section, distances were normalised to have a maximum value of 1, and *Total Coverage *was evaluated in steps of 0.05 between 0 and 1.
